# Elderly road collision injury outcomes associated with seat positions and seatbelt use in a rapidly aging society—A case study in South Korea

**DOI:** 10.1371/journal.pone.0183043

**Published:** 2017-08-11

**Authors:** Yuna Noh, Yoonjin Yoon

**Affiliations:** Department of Civil and Environmental Engineering, Korea Advanced Institute of Science and Technology (KAIST), Yuseong-gu, Daejeon, South Korea; Beihang University, CHINA

## Abstract

**Introduction:**

Aging has long been regarded as one of the most critical factors affecting crash injury outcomes. In South Korea, where the elderly population is projected to reach 35.9% by 2050, the implications of an increasing number of elderly vehicle users on road safety are evident. In this research, the confounding effect of occupant age in a vehicle in terms of seat position and seatbelt use was investigated. In addition, elderly occupants were divided into a younger-old group aged between 65 and 74 years and an older-old group aged 75 years and older in an effort to assess whether the conventional elderly age standard of 65 years should be reconsidered.

**Methods:**

A multinomial logit framework was adopted to predict two-level injury severity using collision data between 2008 and 2015. Predictor variables included gender, age group, seat position, seatbelt, road type, road slope, road surface, road line, and type of vehicle. Five models, a base model with no interactions and four interaction models which were combinations of age group, seatbelt use and seat position, were devised and evaluated.

**Results:**

With no interacting term, age was the most prominent predictor. Elderly occupants were most likely to suffer from severe injury without a seatbelt in all seat positions, and the use of a seatbelt reduced this likelihood the most in the elderly group as well. Front passenger seats had the highest risk to elderly occupants, while the driver seat was statistically insignificant. When the elderly group was divided into the younger-old group and the older-old group, the older-olds were found to be much more vulnerable compared to the younger-olds. In particular, older drivers were five times more likely to suffer a severe injury without a seatbelt.

**Conclusions:**

The degree of injury severity of elderly occupants was reduced the most with the use of a seatbelt, demonstrating the importance of using seat restraints. The sharp increase in the risk of injury of the older-old group suggests that the age standard of 65 years as the elderly group with regard to traffic safety may require reconsideration due to the growing number of elderly vehicle users on the road. Our results provide practical evidence with which to formulate new safety policies, including mandatory seatbelt use, driving age limits and insurance pricing.

## Introduction

According to the United Nations, a society becomes an “aging society” when the number of people aged 65 or older reaches 7% of its total population. It becomes an “aged society” when the proportion of those 65 years or older reaches 14% or more, and a “super-aged society” when those in that age bracket account for more than 20% of the population [1]. Following the UN definition, South Korea became an “aging society” in 2000 and will become an “aged society” in 2018, when 14.4% of its population will be 65 years or older. Korea, one of the most rapidly aging countries in the world, is projected to become a “super-aged society” by 2026 [2, 3]. It is also estimated that the country will have the world’s second oldest population by 2050, with the elderly population reaching 35.9% [4]. Such a projection implies a rapid increase in elderly road users, causing various concerns regarding traffic safety [5]. The aging factor has long been regarded as one of the most critical risk factors affecting injury severity outcomes among vehicle occupant injuries as discussed in Haleem and Gan [6] and reported in work by NIH Senior Health [7].

Traffic safety policies such as mandatory seatbelt use are not fully enforced in South Korea, and the aim of this study was to evaluate whether seatbelt use makes a difference in injury outcomes of the elderly compared to those of other age groups. Seat position was also investigated to determine whether it affects injury severity outcomes for the similar reason that young children are prohibited from sitting in the front passenger seats of vehicles.

There is much literature that discusses the vulnerability of the elderly in auto crashes. Macinko et al. [8] showed that the injury severity from road crashes in the elderly group is higher compared to that of non-elderly groups. Liisa [9], Preusser et al. [10], Lyman et al. [11], Li et al. [12], and Daigneault et al. [13] investigated the association between crash severity and the age factor of older drivers. Previous studies have controlled for various traffic crash factors, including road conditions, crash information, and/or driver information to provide insight into the effects of these factors on the severity level of injuries resulting from a crash.

There has also been much research emphasizing seat position and seat restraints, especially on the outcomes of motor vehicle injuries of children [14–20]. However, there has been a lack of research on elderly occupants with a similar emphasis, and its importance will only increase as South Korea is destined soon to become a super-aged society. To the best of our knowledge, only two previous studies [21,22] have focused on the effect of seat position on elderly vehicle occupants. Smith and Cummings [21] estimated the likelihood of a fatality for various age groups in association with two seat positions (the front and rear seat), seatbelt use, and the presence of airbags. Eluru et al. [22] modeled how various exogenous factors affect the injury severity levels of occupants in different seat positions using a copula-based multivariate method.

Most existing research on this subject [21, 23, 24] classifies seat positions into two groups, front and rear, with a further categorization of the rear seat into outboard and central positions when relevant. However, certain studies [12, 25, 26] have shown that the injury outcomes of crashes involving older drivers, specifically those aged 75 and older, include higher levels of fatality risk. Such results suggest that the front seat should be further categorized into the driver and passenger sides to extract information regarding the driver position separately.

This increasing trend of elderly road users is not only specific to Korea itself but also to other developed and developing countries as well, and several studies have investigated the risk of collisions by the elderly by distinguishing subgroups among the elderly with a cut-off age of 75. Li et al. [12] investigated elderly collision risk and showed that frailty is the overriding factor for increased older driver deaths per mile, while excessive crash involvement, which affects other road users, played a lesser role and becomes apparent by age 75. In another traffic safety study [26], although older drivers have been defined as persons aged 65 years and older, the traffic accident data-based results presented here show that 65- to 74-year-old drivers are relatively safe compared to drivers in their 20s and even compare well to drivers in their 30s. They suggested that older drivers with elevated crash risk levels should be defined as 75 years old and older. Braver and Trempel [25] concluded that when drivers reach the age of 75, there is an upturn in fatality risk, and two thirds of the deaths in crashes involving drivers aged 75 and older were of the drivers themselves, and Rita et al. [27] applied the same cut-off value by grouping the elderly into younger-olds and older-olds in their study.

This study had two research objectives related to the elderly occupants of motor vehicles. First, the confounding effects of seat position and seatbelt use on elderly occupants were analyzed with eight-year road crash data in Seoul, South Korea, between 2008 and 2015. Second, the elderly group was further divided in two age groups, a younger-old group aged between 65 and 74 years and an older-old group aged 75 years and older to assess whether the growing elderly population and changing physical and mental capabilities of the elderly population may necessitate reconsiderations of relevant traffic safety policies. In this study, two injury levels were defined, severe (fatal and major injuries) and non-severe (minor injuries), and three seat positions (driver, front passenger, and rear seat) were considered with an emphasis on older drivers, who are becoming increasingly common. A Multinomial logit model was adopted to compare multiple age groups using odds ratios.

This study helps to raise public awareness about the importance of seatbelt use by elderly road users. Moreover, the accompanying relevant traffic safety regulations could reduce exposure to collision risk by the growing elderly population. In addition, increasing public awareness about the fact that the choice of seat position affects the injury severity outcome could contribute to reducing injury severity levels in collisions involving the elderly. Overall, the results of this study strongly suggest that elderly road users should acquire a better understanding of their risks if involved in a collision and that the almost universal cutoff of 65 years when grouping the elderly requires reconsideration. Immediate attention is needed from both traffic safety agencies and the public, especially in rapidly aging countries such as South Korea.

## Materials and methods

### Data description

The study area was Seoul, which is the capital city of South Korea and which is home to more than one fifth of the nation’s population. It is also among the top six cities for population density worldwide. In 2015, the elderly population comprised 12% of residents in the city [28]. The percentage of road crashes involving elderly occupants in Seoul increased by more than twofold from 3.2% in 2008 to 6.9% in 2015 (see S1 Fig). Recent statistics has shown a steady increase in traffic crashes involving the elderly, and elderly fatalities account for as much as 39% of all traffic crash fatalities [29].

Road crash data from 2008 to 2015 were extracted from the Traffic Accident Analysis System (TAAS), which is managed by the Korea Road Traffic Authority (KoROAD). The authors of this study did not have access to information that could identify individual participants during or after the data collection process.

A total of 160,364 motor crash records were used in our analysis, which included known age, seat position, injury level, and the use of restraints. Infants and toddlers (ages 0–7) were excluded due to mandatory safety seat use for these groups. TAAS records the injury level into four categories: no injury (property damage only: PDO), minor injury, serious injury, and fatal. The PDO category was excluded from our analysis because PDO crash reports contain only driver information.

The three non-PDO injury levels were grouped into two categories—severe (serious and fatal injury) and non-severe (minor injury). After data preparation, 80.0% of all data belonged to the non-severe group, with the remaining 20.0% in the severe group. The ages of 25 and 65 years were adopted as the age standards, as is also adopted commonly in existing injury assessment literature [30–33]. As a result, vehicle occupants were categorized into three age groups: 1) young: 8 to 24 years; 2) middle-aged: 25 to 64 years; and 3) elderly: 65 years or older.

In this study, two sets of analyses were conducted initially: one with two seat categories for the front and rear seats and another with three seat position categories for the driver, front passenger and rear seats. The motivation to examine three seat position categories was so that the front seats could be divided further to extract information regarding the driver position separately from the front passenger seat overall based on previous studies [12, 25, 26] as noted in the introduction section. The results suggested that the front passenger seat and the driver seat have distinct collision risk characteristics and need to be categorized independently. Therefore, three seat positions, the driver seat, the front passenger seat, and the rear seat, were considered in this study.

Table 1 summarizes the distribution of the injury severity according to the seat position and age group. The elderly group has the highest proportion of severe injuries for all seat positions. Note also that the severe injury ratio of the elderly group increased by more than twofold for the front passenger seat and the rear seat when compared to that of the young age group.

**Table 1:** Sample sizes used in the analysis of age and seat position on injury severity.

Age	Seat position/ Injury severity					
	Driver seat		Front passenger seat		Rear seat	
	Non-severe	Severe	Non-severe	Severe	Non-severe	Severe
**Young** (8–24)	2,234 (82.2%)	483 (17.8%)	3,194 (83.4%)	636 (16.6%)	5,935 (85.1%)	1,042 (14.9%)
**Middle-aged** (25–64)	71,794 (80.2%)	19,669 (19.8%)	17,896 (79.9%)	4,511 (20.1%)	21,681 (79.3%)	5,664 (20.7%)
**Elderly** (65+)	3,514 (77.2%)	1,036 (22.8%)	571 (66.2%)	292 (33.8%)	1,397 (63.2%)	815 (36.8%)

### Model variable selection

In addition to age group, seat position, and seatbelt use, the selection of other covariates to be included in the analysis was based on the findings of several previous studies [5, 6, 21, 34–37]. Only the variables for all vehicular occupants that are causally related to the outcome of the injury severity were considered. Therefore, driver-specific information such as ‘sobriety’, ‘violation’, and ‘years of driving experience’ as well as location information such as ‘district’, and time-related information were excluded (see S1 Table for all data fields). All explanatory variables and summary statistics used in the model are shown in Table 2.

**Table 2:** Full description of the explanatory variables and summary statistics.

Variable Characteristic	Variable Name	Summary Statistics
Occupant-related	Age group	F*(young) = 13,524 (*8*.*4%*); *F*(middle-aged) = 139,215 (*86*.*8%*); *F*(elderly) = 7,625 (*4*.*8%*)
	Gender	*F*(male) = 104,183 (*65*.*0%*); *F*(female) = 56,181 (*35*.*0%*)
Vehicle-related	Seat position	*F*(driver seat) = 96,730 (*60*.*3%*); *F*(front passenger seat) = 27,100 (*16*.*9%*); *F*(rear seat) = 36,534 (*22*.*8%*)
	Seatbelt	*F*(on) = 129,287 (*80*.*6%*); *F*(off) = 31,077 (*19*.*4%*)
	Vehicle type	*F*(passenger car) = 135,403 (*84*.*4%*); *F*(van) = 18,145 (*11*.*3%*); *F*(truck) = 6,816 (*4*.*3%*)
Geometric	Road line	*F*(straight) = 151,986 (*94*.*8%*); *F*(curve/bend) = 8,378 (*5*.*2%*)
	Road slope	*F*(downhill) = 14,109 (*8*.*8%*); *F*(non-downhill) = 146,255 (*91*.*2%*)
	Road surface	*F*(dry) = 135,498 (*84*.*5%*); *F*(wet/slippery**) = 24,866 (*15*.*5%*)
	Road type	*F*(intersection) = 68,610 (*42*.*8%*); *F*(non-intersection) = 91,754 (*57*.*2%*)

* *F* = Crash frequency for each involved level (italicized percentages in parentheses)

**wet, deep snow, and freezing merged into wet/slippery

### Multinomial logit model

A multinomial logit model with maximum likelihood estimation was utilized with the R ‘nnet’ package [38]. Estimation was executed with the Newton-Raphson algorithm to perform bias-reducing penalized likelihood optimization. Although alternative models including ordered probit or ordered logit were considered, the multinomial logit framework has several advantages, as described by Washington et al. [39], Savolainen and Mannering [40], Yamamoto et al. [41], Haleem and Gan [6], and Chen et al. [42].

The multinomial logit model was used to reveal the relationships among the outcomes. It contrasts outcomes with a common reference category. It is similar to conducting a series of two-outcome logit models comparing pairs of categories. The model predicts the probability of category membership of a dependent variable based on multiple independent variables. It is assumed that *j* = 1, 2, 3, …, *J*, where *J* denotes the number of categories for the response y (injury severity). Moreover, {π_1_, …, π_*J*_} is denoted as the probabilities of the response categories, satisfying ∑_*j*_ *π*_*j*_ = 1. The multinomial logit model then pairs each of the response categories with a reference category. Assuming “*J*” as the reference category, the possible “*J*−1” logit models are as follows:

$$\eta_{\text{j}}=\text{log}\left(\frac{\pi_{j}}{\pi_{J}}\right)=\alpha_{j}+{Χ}^{T}\beta_{j},\;\;\;\;\;\;\;\;\;\;\;\;j=1,\;2,\;\ldots ,\;J-1$$ (1)

where

α_j_ is the intercept parameter for each of the *J*-1 models,

β_j_ is the vector of the parameter estimates for each of the *J*-1 models, and

**X**^*T*^ is the transpose of the independent variable vector **X**.

The probability of all categories except for the reference category within the response y is estimated as follows:

$$\pi_{\text{j}}=\frac{\text{exp}\left(\beta x\right)}{1+\sum_{j=1}^{J-1}\text{exp}\left(\beta x\right)}$$ (2)

The probability of the reference category “*J*” is estimated as

$$\pi_{\text{J}}=\frac{1}{1+\sum_{j=1}^{J-1}\text{exp}\left(\beta x\right)}.$$ (3)

In this study, the injury severity level was re-classified into two levels (*i*.*e*., *J* = 2), severe and non-severe, and the multinomial logit model was converged to a binary logit model. Statistical significance of the estimates was tested with a 5% significance level. The variance inflation factor (VIF) was calculated to check for multicollinearity among the independent variables.

## Results

### Base model estimation

In Table 3, the odds ratios of each predictor variable, including gender, age group, seat position, seatbelt, road type, road slope, road surface, road line, and type of vehicle, are summarized. The VIF among these variables was 1.01, showing that the predictors are not correlated.

**Table 3:** Estimation results for the model covariates.

Variables	Odds Ratio (OR)	p-value	95% confidence interval (CI)	
Gender				
Male vs. Female	0.808	< .0001	0.788	0.829
Age group				
Young vs. Middle-aged	0.761	< .0001	0.725	0.798
Elderly vs. Middle-aged	1.564	< .0001	1.485	1.646
Seat position				
Front passenger vs. Driver	1.015	0.198	0.981	1.049
Rear vs. Driver	1.048	0.001	1.017	1.079
Seatbelt				
Unrestrained vs. Restrained	1.117	< .0001	1.084	1.152
Road type				
Intersection vs. Non-intersection	1.039	0.001	1.013	1.065
Road slope				
Downhill vs. Non-downhill	1.136	< .0001	1.090	1.185
Road surface				
Wet/slippery vs. Dry	1.096	<0001	1.060	1.133
Road line				
Curved vs. Straight road	1.262	< .0001	1.198	1.329
Type of vehicle				
Truck vs. Passenger car	1.122	< .0001	1.058	1.191
Van vs. Passenger car	1.174	< .0001	1.131	1.219

Among the predictor variables, ‘age group’ yields the most prominent outcome in the elderly group, resulting in the highest odds ratio for a severe injury, while the young group had the lowest odds. The likelihood of a severe injury was also highly affected by gender and seatbelt use. Such findings are analogous to those of Kim et al. [43], Farmer et al. [44], and Neyens and Boyle [45], which found that age, gender, and seatbelt use are closely linked to injury severity.

It was also observed that injury severity was related to the road line, road slope, and road surface, which coincides with the results of various existing studies showing that curved roads, downhill slopes, or wet road surfaces are frequently related to a higher likelihood of a severe injury [33, 36, 46–48].

Note that the effect of seat position was either statistically insignificant or the odds ratio was very close to 1. As presented in the following sections, seat position is a critical factor related to injury severity, especially for the elderly; however, such an effect is not evident when considering the seat position per se.

### Interaction between age group and seatbelt

In Table 4, the estimation results pertaining to the interaction between the age groups and seatbelt use are summarized. As shown in the table, the likelihood of a severe injury for elderly occupants who failed to wear a seatbelt increased by 1.957 times compared with those who were properly restrained. Also note that the middle-aged group showed a relatively small difference in the likelihood of a severe injury with respect to seatbelt use, while there was no statistical significance with regard to seatbelt use for young occupants.

**Table 4:** Estimation results for the interaction between age and seatbelt use.

Variables	OR	p-value	95% CI	
**Young occupant**				
No seatbelt vs. Seatbelt	0.988	0.404	0.899	1.086
**Middle-aged occupant**				
No seatbelt vs. Seatbelt	1.105	< .0001	1.068	1.143
**Elderly occupant**				
No seatbelt vs. Seatbelt	1.957	< .0001	1.754	2.183

### Interaction between age group and seat position

In Table 5, the estimation results for the interaction between age and seat position are summarized. Compared to the middle-aged group, the odds of elderly occupants receiving severe injuries after crashes increased by more than twofold both for the rear (2.233 times) and front passenger (2.029 times) seat. On the other hand, older drivers showed a relatively marginal increase of 20% when compared to middle-aged drivers. A similar comparison between the middle-aged and young age group shows that the probability of a severe injury is reduced in all seat positions with the greatest reduction observed for the rear seat.

**Table 5:** Estimation results for the interaction between age and seat position.

Variables	OR	p-value	95% CI	
**Driver seat**				
Young vs. Middle-aged	0.878	0.005	0.795	0.971
Elderly vs. Middle-aged	1.198	< .0001	1.116	1.286
**Front passenger seat**				
Young vs. Middle-aged	0.790	< .0001	0.721	0.865
Elderly vs. Middle-aged	2.029	< .0001	1.755	2.345
**Rear seat**				
Young vs. Middle-aged	0.672	< .0001	0.625	0.722
Elderly vs. Middle-aged	2.233	< .0001	2.038	2.446

Although the elderly is expected to become more susceptible to crashes due to physical vulnerabilities and slow reflexes, another plausible reason for the universal increase associated with the elderly group here may be related to seat restraint use. This is plausible mainly because mandatory seatbelt laws for all seats in South Korea are only applicable to highways and expressways, and most road segments in cities are exempt. It is also common that mandatory seatbelt use for the front passenger seat is overlooked by elderly occupants.

### Interaction among age group, seat position and seatbelt use

In Table 6, the estimation results for the interaction among age, seat position and seatbelt use are presented. The interaction between seat position and seatbelt use is statistically significant for all combinations except for the case of the driver seat with no seatbelt. Inclusion of seatbelt use notably affected the severity of injuries suffered by elderly passengers, especially for the unbelted elderly in the front passenger seat. When not considering seatbelt use, the likelihood of elderly passengers experiencing a severe injury in the front seat was 2.029, as shown in Table 5. It increased to 2.833 when elderly passengers in the front seat were unbelted and decreased to 1.863 when elderly passengers in the front seat were belted. A comparison with the results in Table 5 further reveals that seat position and seatbelt use have a confounding effect on the severity of injuries to elderly occupants with the estimated odds significantly reduced to 1.742 for the rear seat when the seatbelt is used. A graphical summary of Tables 5 and 6 is included in S2 Fig.

**Table 6:** Estimation results for the interaction between age, seat position, and seatbelt use.

Variables	OR	p-value	95% CI	
**Driver seat** ⅹ **Seatbelt off**				
Young vs. Middle-aged	1.069	0.384	0.686	1.666
Elderly vs. Middle-aged	1.419	0.059	0.916	2.197
**Front passenger seat** ⅹ **Seatbelt off**				
Young vs. Middle-aged	0.792	0.025	0.628	0.999
Elderly vs. Middle-aged	2.833	< .0001	2.026	3.961
**Rear seat** ⅹ **Seatbelt off**				
Young vs. Middle-aged	0.682	< .0001	0.625	0.745
Elderly vs. Middle-aged	2.382	< .0001	2.148	2.643
**Driver seat** ⅹ **Seatbelt on**				
Young vs. Middle-aged	0.867	0.003	0.783	0.961
Elderly vs. Middle-aged	1.195	< .0001	1.112	1.284
**Front passenger seat** ⅹ **Seatbelt on**				
Young vs. Middle-aged	0.787	< .0001	0.713	0.869
Elderly vs. Middle-aged	1.863	< .0001	1.585	2.190
**Rear seat** ⅹ **Seatbelt on**				
Young vs. Middle-aged	0.653	< .0001	0.575	0.742
Elderly vs. Middle-aged	1.742	< .0001	1.433	2.118

### Application to elderly occupants of an age standard of 75 years

The proportion of elderly citizens with an occupation in South Korea is the second highest among the OECD member countries with up to 31.3% of senior citizens actively working, more than twice the OECD average of 14.1% [3]. The report also notes that the employment rate of most working senior citizens aged 65 to 70 years was 44.8%, in contrast to the rate of 17.9% for those aged 75 years and older. It is reasonable to assume that the younger-olds are frequent road users who may have different characteristics affecting injury outcomes compared to the older-olds.

This section presents the estimation results of the severity of injuries of the elderly group after dividing this group into the two subgroups of younger-olds (65 to 74 years old) and older-olds (75 years old and above). As shown in Table 7, the likelihood of older-old occupants suffering a severe injury was significantly higher than that of younger-old occupants for all seat positions when the seatbelt was not worn (off). In particular, older-old drivers who failed to wear a seatbelt were 5.675 times more likely to suffer a severe injury than the middle-aged group. Note that the combination of the driver seat and no seatbelt was statistically insignificant at the 5% level when considering the elderly group as a whole, as shown in Table 6. This is also true for the younger-old group shown in Table 7. The gap between the younger and older-old occupants in the front passenger seat is significant when the seatbelt was not worn (off), although it becomes relatively marginal for the rear seat.

**Table 7:** Estimation results with the further categorization of elderly groups.

	Younger-old (65–74) vs. Middle-aged (N = 6,629)		Older-old (75+) vs. Middle-aged (N = 996)	
	OR (95% CI)	p-value	OR (95% CI)	p-value
**Seatbelt off (%)**	22.2%		48.1%	
Driver seat	1.234 (0.77–1.97)	0.190	5.675 (1.35–23.83)	0.009
Front passenger seat	2.504 (1.71–3.66)	< .0001	4.402 (2.18–8.90)	< .0001
Rear seat	2.198 (1.95–2.48)	< .0001	2.980 (2.46–3.61)	< .0001
**Seatbelt on (%)**	77.8%		51.9%	
Driver seat	1.165 (1.08–1.26)	< .0001	1.693 (1.29–2.23)	< .0001
Front passenger seat	1.874 (1.57–2.24)	< .0001	1.814 (1.24–2.66)	0.001
Rear seat	1.837 (1.47–2.30)	< .0001	1.514 (1.05–2.19)	0.014

The overall odds dropped sharply with the use of seatbelts, especially for the older-old group. In more detail, the odds ratios of the older-old drivers decreased by more than 70%, and the decreases were approximately 60% for the front passenger seat and 50% for the rear seat (See S3 Fig for the graphical results). In other words, the benefit of wearing a seat restraint was greatest for the older-old group. Such a finding is consistent with existing studies by Li et al. [12], Braver and Trempel [25], and Eustace and Wei [26], all of which concluded that when drivers reached age 75, there is an upturn in fatality risk. Our analysis adds to their conclusions that the rate of older-old driver fatalities is greatly affected by seatbelt use.

## Discussion

In this study, the confounding effects of seatbelt use and seat position on injury severity outcomes in an aging society were modeled. Auto crash data over a time period of eight years were collected in Seoul, the capital of South Korea, which is one of the most rapidly aging countries in the world. Injury severity was defined on two levels, severe (fatal and serious injuries) and non-severe (minor injuries), and a multinomial logit model was adopted to predict injury severity levels. Three seat positions, the driver seat, the front passenger seat and the rear seat, were considered.

Among gender, age group, seat position, seatbelt, road type, road slope, road surface, road line, and type of vehicle, age group was the most prevalent predictor of injury severity. When the interaction among age group, seatbelt use and seat position was considered, the elderly group was found most vulnerable to severe injuries when the seatbelt was not worn (off) in the front passenger seat.

The likelihood of a severe injury due to the absence of a seatbelt was highest in the elderly group (Table 4); additionally, the difference in the odds ratios was largest in the elderly group for all seat positions (Table 6).

When the elderly occupants were divided further into younger-olds (65 to 74 years old) and older-olds (75 years old and above), older-old drivers had the greatest likelihood of suffering a severe injury in all cases included in this study. Large gaps between these two elderly groups suggest that the conventional elderly age standard of 65 years may require reconsideration owing to the growing active younger-old population.

One important limitation of our study is that the full effect of the rapidly growing elderly population on road safety requires a broader inclusion of other crash categories, such as elderly pedestrian and bicycle injuries. Although the nature of the data collected here prevented us from including such crash types, the authors believe that this vehicle occupant study addresses the majority of road crashes, and the conclusion drawn from our study could characterize the overall trend in a rapidly aging society. Other protective measures such as airbags were also excluded in this study because the penetration of airbags in passenger cars is nearly 100% in South Korea.

Because South Korea is in the process of enacting mandatory seatbelt use for all seat positions as of late 2017, a before-and-after study using a time-series model with an intervention method such as the intervention ARIMA is planned in the near future to assess the effectiveness of such a policy on the elderly population.

## Conclusions

The vulnerability of elderly occupants in vehicles varies greatly depending on their seat position and seatbelt use, suggesting that the proper enforcement of the use of safety restraints may play an important role in reducing elderly injuries in road crashes. The clear distinction between the two elderly groups examined in this study, termed the younger-olds and the older-olds, indicates that safety policies should acknowledge the changing physical and mental capabilities of the elderly, with more adaptive approaches in an effort to address the rapidly growing number of elderly vehicle users.

## Supporting information

S1 Fig Percentage of road crashes involving elderly occupant in Seoul, South Korea between 2008 and 2011. (TIF)

S2 Fig Severe injury odds ratios of the young and elderly age group by seat positions and seatbelt use with the middle-aged group as a baseline. (TIF)

S3 Fig Severe injury odds ratios of the younger and older elderly groups by seat positions and seatbelt use with the middle-aged as a baseline. (TIF)

S1 Table TAAS meta data. (PDF)
