# Supplementary figures and images for: Elderly road collision injury outcomes associated with seat positions and seatbelt use in a rapidly aging society—A case study in South Korea
Source: PLoS One. 2017 Aug 11;12(8):e0183043. doi: 10.1371/journal.pone.0183043 (PMC5553646; doi:10.1371/journal.pone.0183043)

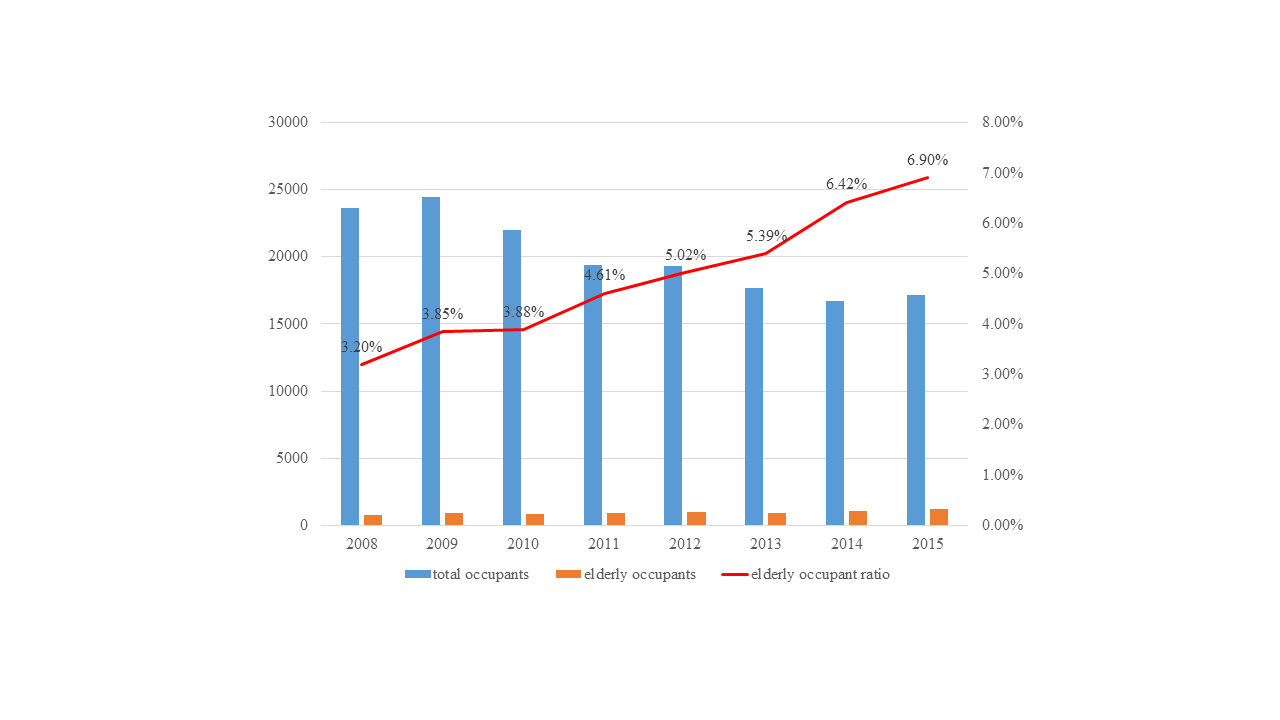

Supplement: S1 Fig — (TIF) [file pone.0183043.s001.tif]

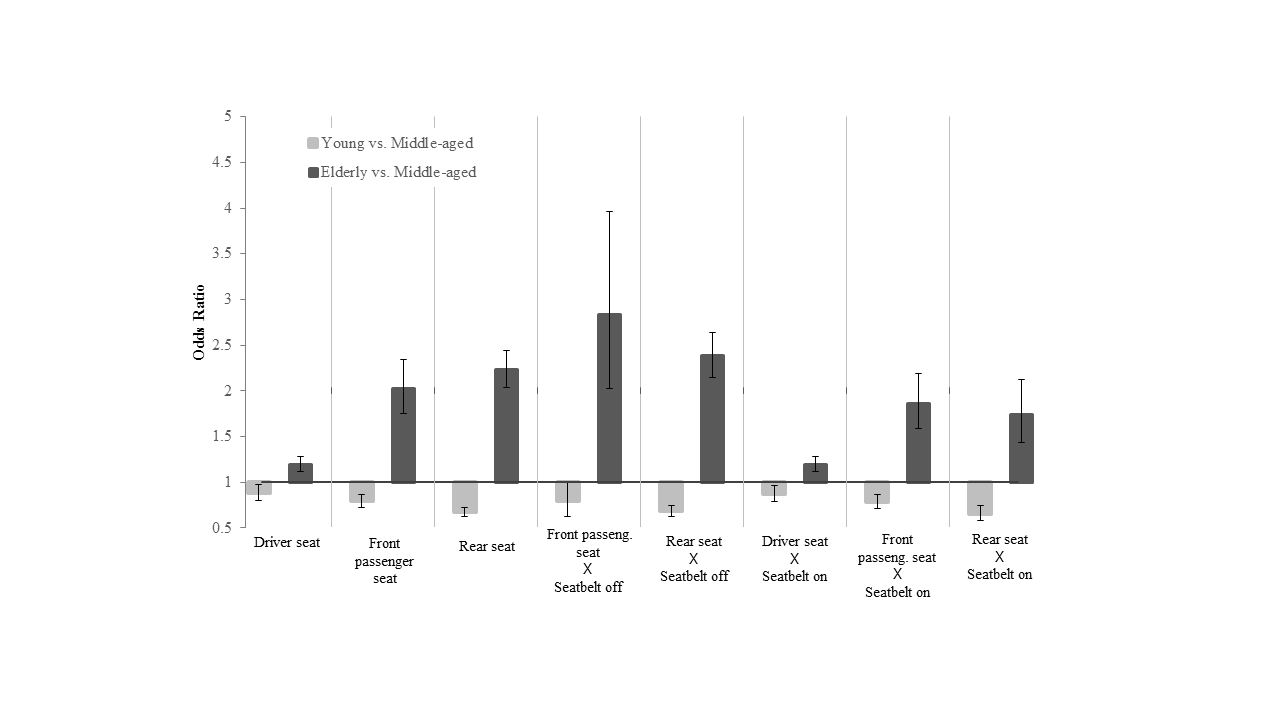

Supplement: S2 Fig — (TIF) [file pone.0183043.s002.tif]

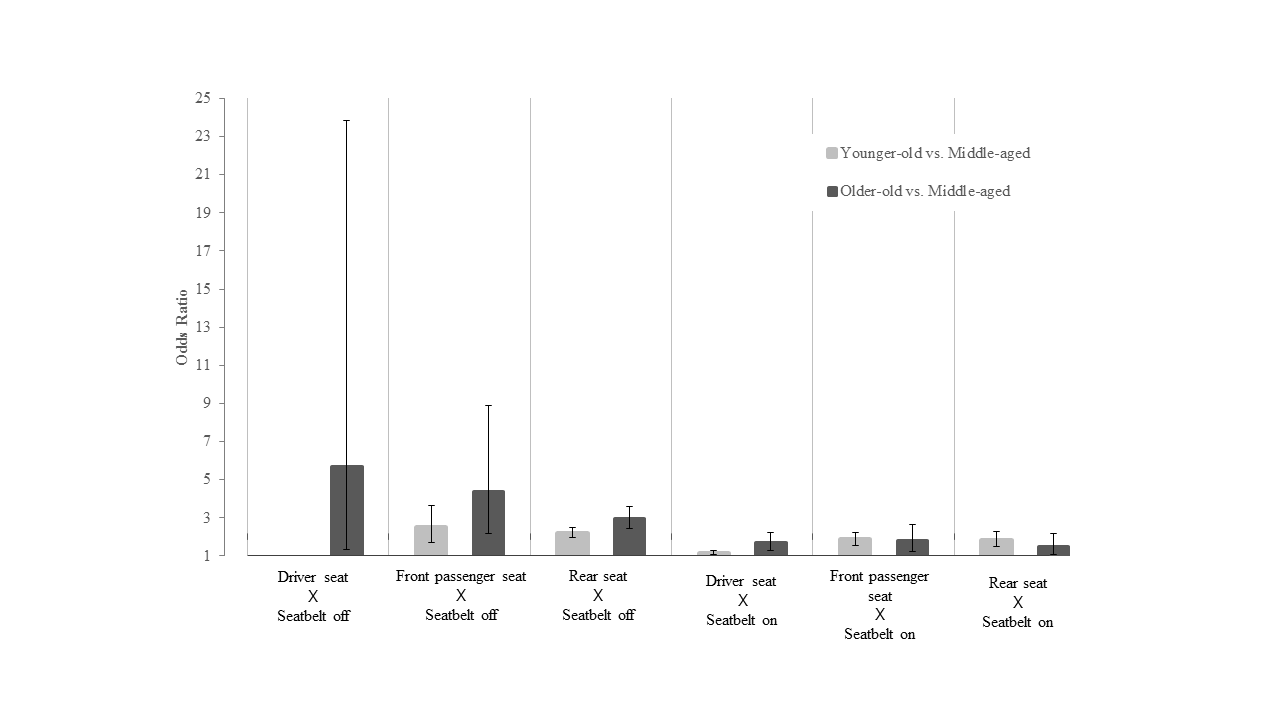

Supplement: S3 Fig — (TIF) [file pone.0183043.s003.tif]
