# Supplementary material for: Elderly road collision injury outcomes associated with seat positions and seatbelt use in a rapidly aging society—A case study in South Korea
Source: PLoS One. 2017 Aug 11;12(8):e0183043. doi: 10.1371/journal.pone.0183043 (PMC5553646; doi:10.1371/journal.pone.0183043)
Supplement: S1 Table — (PDF) [file pone.0183043.s004.pdf]

Data Field Type	Data Field	Values
Occupant-related	Age	1 - 98
	Gender	Male; Female
	Injury severity	Fatal; Serious injury; Minor injury; Property Damage Only (PDO); Unknown
Geometric	Road type	Intersection; Road; Crosswalk; Overpass; Underground road; Unknown
	Road line	Straight; Curve; Others
	Road surface	Dry; Wet; Deep snow; Frozen; Others
	Road slope	Flat; Uphill; Downhill; Others
Vehicle-related	Vehicle type	Passenger car; Bus; Van; Truck; Motorcycle; Construction vehicle; Agricultural vehicle; Unknown; Others
	Seat position	Driver seat; Front passenger seat; Rear seat; Unknown
	Seatbelt	Restrained; Unrestrained; Unknown
Driver-related	Type of driver's license	Type 1: Large vehicles; Type 2: Small vehicles; International driver license; Practice license; No license; Unknown
	Years of driving experience	Less than 1 year; Less than 2 years; Less than 3 years; Less than 4 years; Less than 5 years; Less than 10 years; Less than 15 years; More than 15 years; Unknown
	Violation	Speed violation; Disobeying signal; Improper passing; Improper railroad crossing; Sidewalk violation; Driving with doors open; Center line crossing and division violation; Impeding pedestrians on the crosswalk; School zone; Others
	Sobriety	00; 0.05-0.09%; 0.10-0.14%; 0.15-0.19%; 0.20-0.24%; 0.25-0.29%; 0.30-0.34%; More than 0.35%; Impossible to measure; Measurement failure; Unknown
	Movement	Proceeding straight; Stopped; Making U-turn; Passing other vehicle; Making right/left turn; Changing lanes; Backing; Others
Time-related	Year	2008; 2009; 2010; 2011; 2012; 2013; 2014; 2015
	Month	Jan.; Feb.; Mar.; Apr.; May.; Jun.; Jul.; Aug.; Sep.; Oct.; Nov.; Dec.
	Day of week	Monday; Tuesday; Wednesday; Thursday; Friday; Saturday; Sunday
Location	District	Dobong-gu; Dongdaemun-gu; Dongjak-gu; Eunpyeong-gu; Gangbuk-gu; Gangdong-gu; Gangnam-gu; Gangseo-gu; Geumcheon-gu; Guro-gu; Gwanak-gu; Gwangjin-gu; Joongnang-gu; Jongno-gu; Jung-gu; Mapo-gu; Nowon-gu; Seocho-gu; Seodaemun-gu; Seongbuk-gu; Seongdong-gu; Songpa-gu; Yangcheon-gu; Yeongdeungpo-gu; Yongsan-gu

Other environment	Weather	Clear; Cloudy; Snow; Rain; Fog; Others
